# The Impact of Homogenization on Donor Human Milk and Human Milk–Based Fortifiers and Implications for Preterm Infant Health

**DOI:** 10.1093/cdn/nzab147

**Published:** 2021-12-08

**Authors:** Sarah M Reyes, Biranchi Patra, Melinda J Elliott

**Affiliations:** Prolacta Bioscience®, Duarte, CA, USA; Prolacta Bioscience®, Duarte, CA, USA; Prolacta Bioscience®, Duarte, CA, USA; Pediatrix Medical Group of Maryland, Rockville, MD, USA

**Keywords:** human milk, homogenization, exclusive human milk diet, EHMD, milk fat globule membrane, infant development, neurodevelopment, bioactive proteins, premature infants

## Abstract

An exclusive human milk diet (EHMD) has been shown to reduce health complications of prematurity in infants born weighing ≤1250 g compared with cow milk–based diets. Accordingly, the number of available human milk (HM)-based nutritional products continues to increase. Newly available products, and those reportedly soon to enter the market, include homogenized donor HM and homogenized HM–based fortifiers. Existing literature demonstrating the benefits of an EHMD, however, is limited to *non*-homogenized HM-based products. Herein, we summarize existing evidence on the impact of homogenization on HM, with a particular focus on changes to the macromolecular structure of the milk fat globule and the subsequent impact on digestion kinetics. We use these published data to create a conceptual framework for the potential implications of homogenized HM-based nutritional products on preterm infant health. Importantly, we underscore that the safety and efficacy of homogenized HM-based products warrant investigation.

## Teaser

Human milk (HM)-based nutritional products are not created equally. Homogenization alters the evolutionarily evolved structure of HM, yet safety and efficacy research are lacking for homogenized HM-based nutritional products.

## Introduction

The best source of nutrition for premature infants born weighing <1500 g is their mothers’ own milk (MOM) fortified appropriately with protein, minerals, and vitamins; when MOM is unavailable or contraindicated, donor human milk (DHM) should be substituted (1). Human milk (HM)-based fortifiers added to MOM or DHM—an exclusive human milk diet (EHMD)—has shown promising results in clinical studies to reduce health complications of prematurity in infants weighing ≤1250 g compared with traditionally used cow milk–based fortifiers or preterm infant formulas, especially for necrotizing enterocolitis (NEC) (2–9).

The benefits derived from an EHMD diet are 2-fold: delivering the nutrition required to offset missed nutrient accretion that occurs during the third trimester and reducing the complications associated with enteral feeding (2, 10, 11). Before the advent of HM-based fortifiers, MOM or DHM was fortified with cow milk–based fortifiers. However, preterm infants fed cow milk–based fortifiers had higher risk of NEC and severe retinopathy of prematurity despite having a base diet of HM (8, 12). A combined analysis of 2 randomized clinical studies demonstrated that for every 10% increase in the volume of milk containing cow milk–based protein, the risk of NEC increased by 11.8%, surgical NEC by 21%, and sepsis by 17.9% (7). Unsurprisingly, the adoption of HM-based nutritional products is increasing in hospitals across the United States and globally.

As the use of HM-based nutritional products grows, so do the number of products available in the market. Currently available products include DHM, HM-based multinutrient fortifiers, and HM-based caloric fortifiers. These products are used in combination with MOM and/or DHM under the direction of health care professionals to meet the individual nutritional needs of the most medically fragile infants (2–8, 11, 12). Importantly, however, not all HM-based nutritional products are created equally. The manufacturing processes used to produce DHM and HM-based fortifiers vary widely among the manufacturers. These processes influence the composition of HM-based nutritional products, and possibly their efficacy. For example, currently available products undergo heat-treatment (e.g., pasteurization) to ensure they are safe for premature infants to consume. However, different heat treatments result in highly variable losses to important biologically active components including anti-infective properties, such as immunoglobulins, lysozyme, and lactoferrin (13–16). Therefore, it is prudent that physicians, nurses, and hospital administrators involved in purchasing decisions consider manufacturing processes when deciding which HM-based nutritional products to use for their neonatal intensive care unit (NICU) patients.

Newly available products, and those reportedly soon to enter the market, include homogenized DHM and homogenized HM-based fortifiers (17, 18). Herein, we discuss homogenization: what it is, why it is performed, and what evidence supports its use for HM-based nutritional products designed for ill newborn and premature infants. We propose a conceptual framework for the potential implications of homogenized HM-based nutritional products on preterm infant health, highlighting areas of research needed to establish their safety and efficacy.

## Homogenization and its Impact on HM Composition and Digestion Kinetics

### What is homogenization?

Homogenization is a physical process that evenly disperses 2 mutually nonsoluble liquids to create a single uniform mixture. Milk is homogenized to disperse fat droplets and prevent the cream from rising to the top. In the dairy industry, homogenization is used to improve cow milk's taste, consistency, and appearance as well as to extend its shelf-life (19).

### Why are some HM-based nutritional products homogenized?

Some newly available and upcoming HM-based nutritional products are homogenized, although the rationale for homogenization is unclear. The strongest rationale is based on the hypothesis that homogenization may improve weight gain of very-low-birth-weight premature infants. This hypothesis gained traction after evidence showed that fecal excretion of fat is lower in infants fed homogenized HM than in those fed nonhomogenized HM (20). These data suggest that fat absorption is higher in infants fed homogenized HM. Even so, there is currently no clinical evidence demonstrating that this translates into improved or more appropriate growth outcomes in premature infants. In contrast, HM-based nutritional products produced from nonhomogenized HM have been shown in clinical studies to result in adequate growth without long-term insulin resistance and excess adiposity in infants born weighing ≤1250 g (11, 21–24).

Another rationale for homogenizing HM-based nutritional products may be to reduce fat loss during enteral feeding. Continuous enteral feeding can lead to fat loss of HM because the fat sticks to plastic tubing (25). Homogenization reduces the size of the milk fat globule, which may reduce its adherence to the tubing (26, 27). Even so, feeding homogenized HM-based nutritional products is unnecessary because fat loss from HM is already mitigated by adding nonhomogenized HM-based fortifiers or HM cream (28). In addition, nonhomogenized HM cream can be used to “prime” the tubing before enteral feeding to further minimize fat loss (29).

More likely, the rationale for homogenizing HM-based nutritional products may be to counter the effects of high-temperature processing, such as retort sterilization or ultra-high temperature (UHT) processing. Retort sterilization is a heat treatment common in canned food manufacturing during which milk is exposed to high temperatures and pressure to eliminate viral and bacterial pathogens. UHT processing is a higher heat treatment for shorter duration than retort sterilization. New and upcoming homogenized HM-based nutritional products also undergo either retort sterilization or UHT processing. Unfortunately, high-temperature processing methods can lead to loss of fat content in HM because they cause the fat to adhere to the container. Sterilization of HM resulted in loss of >10% of fat content, whereas pasteurization of HM had no effect on its fat content (30). It is plausible that homogenization is used to uniformly distribute the fat content back into solution.

Ultimately, the rationale for homogenizing HM-based nutritional products is based on the weak scientific premise that it is advantageous for infant weight gain, although it is more likely performed to counter the negative effects of harsh heat treatments on HM fat.

### Effects of homogenization on milk composition

Homogenization affects the properties of milk in a variety of ways. The most serious of these relate to changes in the macromolecular structure of the milk fat globule. Specifically, homogenization removes a large proportion of the outer membrane, resulting in smaller milk fat droplets to which milk serum proteins adsorb (31). This change in the macromolecular structure of the milk fat globule can alter digestion kinetics and the bioavailability of nutrients, especially proteins, fats, and fat-soluble nutrients. The implications of such changes on preterm infant health outcomes have been inadequately investigated.

**FIGURE 1:**
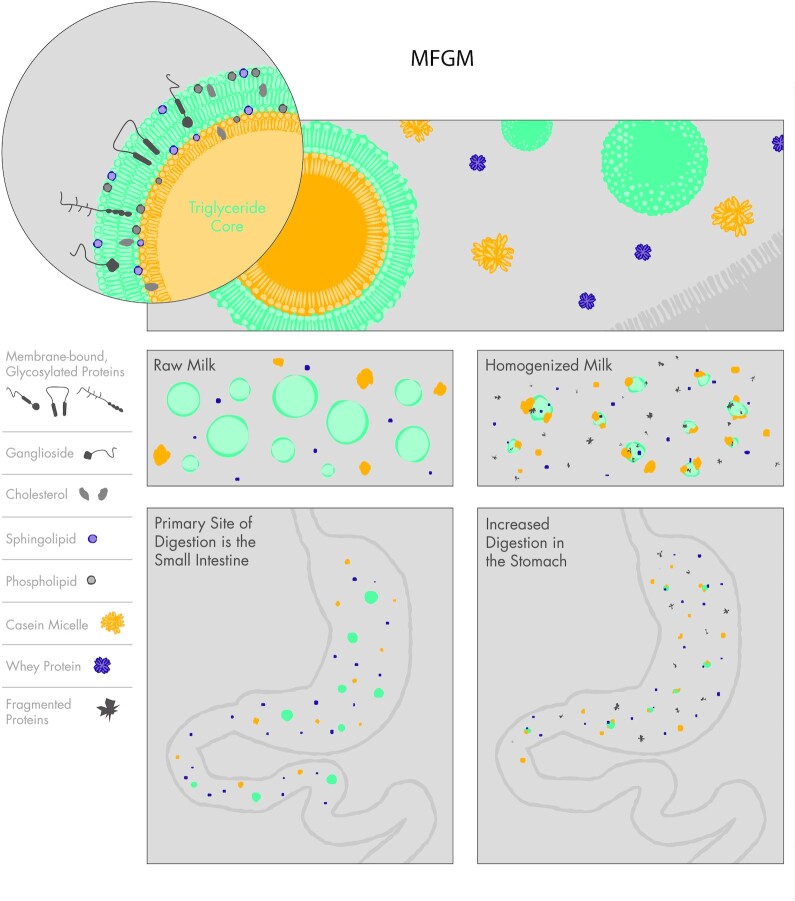
The impact of homogenization on human milk fat globules. Homogenization disrupts the MFGM, leading to adsorption of milk serum proteins, including whey proteins, casein micelles, and casein micelle fragments. The resulting smaller fat droplets coated with milk serum proteins interact differently with digestive enzymes such as gastric and pancreatic lipases, rendering them more susceptible to premature digestion in the stomach. MFGM, milk fat globule membrane.

The milk fat globule is the primary vesicle that delivers fat and fat-soluble nutrients to the infant (32). The native milk fat globule consists of an inner core of triglycerides surrounded by a lipid bilayer derived from a plasma membrane, called the milk fat globule membrane (MFGM) (**Figure 1**). The MFGM contains numerous embedded lipids, proteins, and carbohydrates including sphingolipids, phospholipids, proteins, glycoproteins, gangliosides, and cholesterol that are important for protection against pathogens, maturation of the infant gut, and the development of the immune, metabolic, and central nervous systems (33). The structure of the MFGM and the roles of its various components have been reviewed in detail (31, 33–35).

Homogenization disrupts the MFGM, exposing the hydrophobic lipid core to the surrounding aqueous environment (31, 36). Whey protein, casein micelles, and casein micelle fragments subsequently adsorb to the exposed lipid core, leading to smaller fat droplets coated with milk serum proteins (Figure 1). In preclinical studies, homogenization-induced changes to the physical and chemical properties of the milk fat globules alter their interactions with digestive enzymes, including gastric and pancreatic lipases, and render them more susceptible to premature digestion in the stomach (37–40) (Figure 1). An in vivo pilot study confirmed these preclinical findings. In a clinical trial, premature infants fed homogenized HM presented with higher levels of gastric lipolysis and proteolysis, and delayed gastric emptying, compared with infants fed nonhomogenized HM (41). Together, these findings suggest that homogenized HM could increase the amount of fatty acids available for absorption (20) and have a positive impact on weight gain. Nevertheless, this study was small (it only included 8 infants) and thus was not designed to evaluate important clinical outcomes including growth, feeding intolerance, or neurodevelopmental outcomes. It is unclear if this possible increased weight gain is at the expense of increased feeding intolerance and decreased availability of bioactive lipid components necessary for brain growth.

## Clinical Implications of Feeding Homogenized HM-Based Products to Preterm Infants and Avenues for Needed Research

### Potential clinical implications of a disrupted MFGM

The MFGM and its various components have been implicated in favorable neurodevelopmental outcomes and low risk of infections and feeding intolerance (33), and are generally lacking in commercial preterm infant formulas. Thus, the MFGM has been the subject of much interest in recent years as scientists endeavor to understand the biological origins explaining the more favorable outcomes observed in breastfed infants than in formula-fed infants.

**FIGURE 2:**
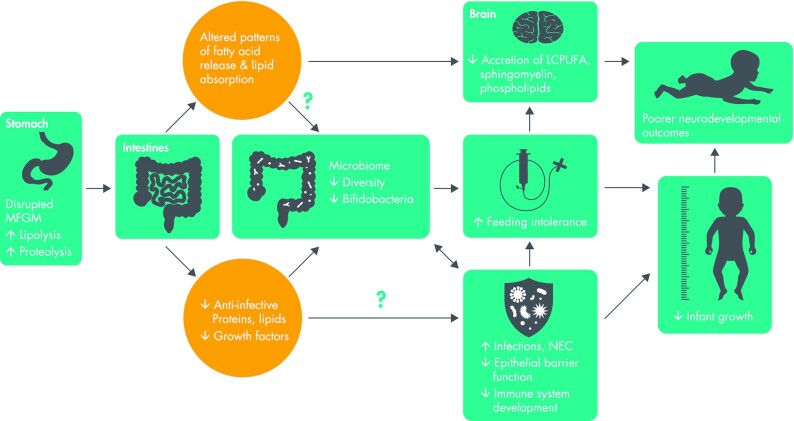
Conceptual framework of the potential implications of homogenized HM-based nutritional products on health outcomes of premature infants. LCPUFA, long-chain PUFA; MFGM, milk fat globule membrane; NEC, necrotizing enterocolitis.

In vivo studies of formula-fed infants support the hypothesis that the MFGM may contribute to the benefits of HM-feeding. For instance, the addition of cow or whey-sourced MFGM to infant formula has been shown in clinical trials to result in fewer infections and improved neurodevelopment outcomes compared with standard, MFGM-free formulas (42–45). Nevertheless, the benefits of HM may be more than the sum of all parts. Rather, the structural properties of HM were evolutionarily evolved to promote optimal health of the recipient infant (31). It has been suggested that this structure influences the rate of digestion and timing of absorption of the various components, and that this may have an important role in the health-promoting benefits of HM (31). If so, it is plausible that homogenization may influence the bioavailability and bioactivity of MFGM components and, in turn, their benefits to infant health. We created a conceptual framework to visualize the potential implications of a disrupted MFGM for several clinically significant outcomes in premature infants (**Figure 2**). This conceptual framework represents important areas of needed research.

### Neurodevelopment outcomes

The effects of a disrupted MFGM on the neurocognitive benefits of HM for preterm infants is a critically important area of research in need of investigation (Figure 2). Premature infants are at risk of long-term poor neurocognitive and academic outcomes (46). However, premature infants fed HM experience a lower incidence of long-term neurodevelopmental disabilities and more favorable neurodevelopmental outcomes than those fed preterm infant formulas (1).

The neurocognitive benefits of HM are attributed, in part, to the numerous HM components important for brain development, including sialic acid, gangliosides, sphingomyelin, choline, cholesterol, and PUFAs (33). These components are naturally resistant to gastric digestion in the stomach owing to the macromolecular structure of the intact milk fat globule. They are delivered intact to the small intestine, the main site for digestion and absorption of lipids (47). Through complex interactions with pancreatic and biliary secretions the intact milk fat globule is broken down and its lipids absorbed (47). When milk is homogenized and the MFGM is disrupted, more of these lipids are broken down prematurely in the stomach (41). The clinical significance of their premature digestion has not been adequately investigated.

In addition to causing premature digestion of HM lipids, homogenization can alter the accumulation patterns of free fatty acids. Fatty acids are a normal part of the inner triglyceride core of the milk fat globule. Three fatty acids connected to a glycerol molecule make up a single triglyceride. Typically, long-chain fatty acids are released before SCFAs so they can be absorbed in the small intestine (47). However, ex vivo models of digestion demonstrated that homogenized cow milk had early release of SCFAs and delayed release of long-chain fatty acids compared with nonhomogenized cow milk (38). It is unclear whether delayed release of long-chain fatty acids negatively affects their absorption. This is an important avenue for future research because it may influence accumulation of long-chain fatty acids in the brain and neurocognitive outcomes in very-low-birth-weight preterm infants.

Long-chain fatty acids, especially long-chain PUFAs (LCPUFAs), are important for brain development. LCPUFAs, including DHA (22:6n–3), are primarily accumulated in the fetal brain during the third trimester (48).

Achieving optimal accretion of LCPUFAs in the brain of infants born prematurely remains a serious clinical challenge, although recent evidence suggests feeding an EHMD using nonhomogenized HM-based fortifiers may be beneficial. Among premature infants fed fortified HM, those fed HM with a nonhomogenized HM-based fortifier maintained normal blood concentrations of DHA in the first 3 wk of life compared with a 30% decline in infants fed HM fortified with a homogenized cow milk–based fortifier (49).

These results are intriguing because all infants in this study were fed HM (>70% MOM), but 1 group received a nonhomogenized HM-based fortifier with intact MFGM and the other group received a homogenized cow milk–based fortifier, lacking MFGM. Further investigation of the effect of a disrupted MFGM structure on blood DHA concentrations should be undertaken before homogenization is deemed appropriate for HM-based fortifiers.

### Epithelial barrier function and risk of infections

New and upcoming homogenized HM-based nutritional products also undergo high heat processing, such as UHT or retort sterilization—a combination that plausibly could have implications for infant epithelial barrier function and risk of infection and NEC (Figure 2). An MS-based protein determination study reported that DHM that underwent homogenization and retort sterilization had significantly lower concentrations of several immune-modulating proteins, including IgA, IgG, lysozyme, lactoferrin, α-lactalbumin, α-antitrypsin, and osteopontin, than nonhomogenized DHM that was processed by vat or Holder pasteurization (13). In addition, in an ex vivo model of digestion, cow milk that underwent homogenization and UHT had significantly lower concentrations of intact whey proteins, IgG, and lactoferrin than cow milk that underwent homogenization alone (50). Taken together, these data suggest that homogenized HM-based nutritional products that undergo UHT or retort sterilization could deliver fewer anti-infective lipids and proteins and fewer growth factors to the infant gastrointestinal tract. If so, this could delay maturation of the epithelial barrier, which is required to prevent bacterial translocation (51, 52), and it could increase the risk of secondary infections, late-onset sepsis, or NEC, especially in infants exposed to antibiotics (53, 54). These possibilities also warrant investigation.

### Feeding tolerance and growth outcomes

Feeding tolerance and growth are critically important clinical outcomes for preterm infants, and the role of the microbiome in these outcomes has gained attention (55–57). A recent study found that gut microbiome richness was positively associated with feeding tolerance and growth among exclusively HM-fed preterm infants (57). These results support the possibility that the microbiome may be one of the biological mechanisms underlying feeding tolerance and growth—another area needing further investigation.

It has been suggested that the macromolecular structure of the milk fat globule enables advantageous absorption (which may not necessarily be rapid or complete) that optimizes lipid tissue distribution (31, 58). Specifically, although it is well established that changes in the physiochemical properties of milk fat globules influence digestion and absorption of nutrients in non-HM products (59, 60), the food structure is a complex matrix of diverse components designed to be digested and absorbed during specific phases of digestion. This multiphasic digestion of the native milk fat globule influences the protein–protein interactions, such as the interaction of MFGM proteins and probiotic bacteria, *Bifidobacterium* spp., and lactic acid bacteria (61). These interactions are important to the survival and adhesion of probiotic bacteria in the gastrointestinal tract as well as the development of mucosal immunity (61). This may explain the positive association between gut microbiome richness and feeding tolerance and growth outcomes in very-low-birth-weight infants (57). The possibility that homogenized HM-based nutritional products alter this biological mechanism and, in turn, feeding tolerance and growth merits investigation (Figure 2).

### Safety and efficacy of homogenized HM-based nutritional products are unclear

In summary, homogenization changes the macromolecular structure and composition of the milk fat globule in ways that influence protein and fat bioavailability and bioactivity. Although preliminary studies indicate that this may increase fat absorption (20, 62, 63), the implications for several clinically relevant outcomes including infection, NEC, neurodevelopment, feeding intolerance, and growth have not been studied (Figure 2). As HM scientists and medical professionals, it is our opinion that the use of industrial processing techniques such as homogenization with or without high heat processing should be avoided for HM-based nutritional products until their safety and efficacy have been established.

## Concluding Remarks

HM-based nutritional products as part of an EHMD have been shown in clinical studies to improve health and reduce the complications of prematurity in very-low-birth-weight infants. Accordingly, the use of these products and EHMD protocols are increasing globally as are the number of available products. Some new and upcoming products are homogenized which alters MFGM structure, a complex mixture of proteins, lipids, and carbohydrates shown to be important in infection, neurodevelopment, and growth. Changes to the macromolecular structure of the milk fat globule have unknown consequences for the health of premature infants. Thus, investigation of homogenization-induced changes to the macromolecular structure and function of the milk fat globule is critical to establish the safety and efficacy of homogenized HM-based nutritional products, especially those that also undergo high heat treatments including retort sterilization and UHT processing. Importantly, the published clinical benefits of HM-based nutritional products only apply to currently available nonhomogenized products. The safety and efficacy of homogenized HM-based nutritional products have not been established.

Box 1 **Key Messages**

- Traditionally used in the dairy industry, homogenization is used to improve cow milk's taste, consistency, and appearance as well as to extend its shelf-life.
- Homogenization disrupts the milk fat globule, which may lead to premature digestion of bioactive components linked to favorable neurological outcomes, including sphingolipids and long-chain PUFAs. The implications of these changes for infant health are unknown.
- The described clinical benefits of human milk (HM)-based nutritional products only apply to currently available nonhomogenized products. The safety and efficacy of homogenized HM-based nutritional products have not been established.

## Data Availability

No new data were generated or analyzed in support of this research.

## Notes

Supported by Prolacta Bioscience (to SMR). The authors are employed by Prolacta Bioscience, and are completely responsible for the design, implementation, analysis, and interpretation of the data.

Author disclosures: SMR, BP, and MJE are employees of Prolacta Bioscience. Prolacta Bioscience is a supplier of nonhomogenized human milk-based products for premature infants.

Abbreviations used: DHM, donor human milk; EHMD, exclusive human milk diet; HM, human milk; LCPUFA, long-chain PUFA; MFGM, milk fat globule membrane; MOM, mothers’ own milk; NEC, necrotizing enterocolitis; UHT, ultra-high temperature.
